# Granzyme B Cleaves Decorin, Biglycan and Soluble Betaglycan, Releasing Active Transforming Growth Factor-β1

**DOI:** 10.1371/journal.pone.0033163

**Published:** 2012-03-30

**Authors:** Wendy A. Boivin, Marlo Shackleford, Amanda Vanden Hoek, Hongyan Zhao, Tillie L. Hackett, Darryl A. Knight, David J. Granville

**Affiliations:** 1 UBC James Hogg Research Centre, Institute for Heart+Lung Health, St. Paul's Hospital, Vancouver, British Columbia, Canada; 2 Department of Pathology and Laboratory Medicine, University of British Columbia, Vancouver, British Columbia, Canada; 3 Department of Anesthesiology, Pharmacology and Therapeutics, University of British Columbia, Vancouver, British Columbia, Canada; Leiden University Medical Center, The Netherlands

## Abstract

**Objective:**

Granzyme B (GrB) is a pro-apoptotic serine protease that contributes to immune-mediated target cell apoptosis. However, during inflammation, GrB accumulates in the extracellular space, retains its activity, and is capable of cleaving extracellular matrix (ECM) proteins. Recent studies have implicated a pathogenic extracellular role for GrB in cardiovascular disease, yet the pathophysiological consequences of extracellular GrB activity remain largely unknown. The objective of this study was to identify proteoglycan (PG) substrates of GrB and examine the ability of GrB to release PG-sequestered TGF-β1 into the extracellular milieu.

**Methods/Results:**

Three extracellular GrB PG substrates were identified; decorin, biglycan and betaglycan. As all of these PGs sequester active TGF-β1, cytokine release assays were conducted to establish if GrB-mediated PG cleavage induced TGF-β1 release. Our data confirmed that GrB liberated TGF-β1 from all three substrates as well as from endogenous ECM and this process was inhibited by the GrB inhibitor 3,4-dichloroisocoumarin. The released TGF-β1 retained its activity as indicated by the induction of SMAD-3 phosphorylation in human coronary artery smooth muscle cells.

**Conclusion:**

In addition to contributing to ECM degradation and the loss of tissue structural integrity *in vivo*, increased extracellular GrB activity is also capable of inducing the release of active TGF-β1 from PGs.

## Introduction

Granzyme B (GrB) is a pro-apoptotic serine protease initially characterized in the granules of CTLs and NK cells. GrB is released towards target cells along with the pore-forming protein, perforin, resulting in its perforin-dependent internalization into the cytoplasm and subsequent induction of apoptosis [1], [2]. However, while once thought to be primarily involved in CTL/NK cell-mediated apoptosis, it is now recognized that GrB can be expressed by other inflammatory cells (eg. macrophages, mast cells, dendritic cells, basophils) as well as non-immune cells (eg. chondrocytes, smooth muscle cells, keratinocytes, reviewed in [3]). As non-immune cells do not possess cytotoxic granules, rarely express perforin and do not readily form immunological synapses with target cells, GrB is secreted from these cells into the extracellular milieu. Indeed mounting evidence suggests that GrB exhibits non-apoptotic, perforin-independent roles involving matrix degradation in the pathogenesis of chronic inflammatory diseases (Summarized in [2], [3]).

Extracellular levels of GrB are elevated in the bodily fluids in chronic inflammatory diseases such as atherosclerosis, COPD and rheumatoid arthritis [2], [4], [5], [6], [7]. As further direct support for an extracellular role for GrB in disease, we have recently shown that GrB contributes to murine abdominal aortic aneurysm (AAA) pathogenesis through the cleavage of extracellular matrix (ECM) proteins such as fibrillin-1 and decorin [8], [9]. We have also shown that GrB-cleavage of decorin contributes to collagen disorganization and frailty in the skin of aging mice [10]. In the latter study, GrB deficiency attenuated collagen disorganization, reduction of thick collagen bundles and skin thinning.

Although extracellular GrB accumulation in chronic inflammation is well-established (Reviewed in [2]), the consequences of active GrB accumulation in the extracellular milieu requires further investigation. In addition to fibrillin-1, known extracellular GrB substrates include aggrecan, von willebrand factor, plasmin, fibronectin, laminin, vitronectin, and neuronal glutamate receptor [8], [11], [12], [13], [14], [15], [16], however the known pathophysiological implications of ECM cleavage are limited.

The ECM is a fundamental component of tissue, providing a structural framework for stability and elasticity. It also provides an essential scaffold for cell survival, acts as a molecular filter, and influences cell signaling and phenotype. In particular relevance to the present study, the ECM acts as a reservoir for growth factors and cytokines by influencing their storage, location, concentration, activation, synthesis and degradation [17]. Extracellular proteases such as the matrix metalloproteases (MMPs) act in regulating growth factor bioavailability by cleaving ECM and releasing sequestered cytokines, as well as by activating latent growth factors [17], [18]. One such family of growth factors is TGF-β, which is involved in numerous processes including differentiation, migration and ECM synthesis [19], [20]. TGF-β is normally secreted as a latent protein but both the active and inactive forms of TGF-β are bound and sequestered by ECM, albeit by different mechanisms. For example, fibrillin-1 sequesters inactive TGF-β by interacting with the latent TGF-β binding protein (LTBP) while active TGF-β can bind directly to the ECM proteoglycans (PGs) decorin, biglycan and soluble betaglycan [17], [21], [22], [23].

Decorin and biglycan are members of the small leucine rich PG family and are involved in the sequestration of active TGF-β and in collagen fibre spacing [23], [24], [25]. Betaglycan, also known as TGF-β receptor III, is a PG receptor involved in TGF-β signaling. Its extracellular domain acts as a soluble receptor following cleavage by membrane type-matrix metalloproteinase (MT-MMP1), in a process known as ectodomain shedding [26], [27]. This soluble betaglycan resides in the ECM and functions in TGF-β sequestration [22], [28].

In the present manuscript we demonstrate that GrB cleaves decorin, biglycan and betaglycan and identify sites of proteolysis. We show that the released TGF-β1 is active and induces the phosphorylation of SMAD-3 in human coronary artery smooth muscle cells (HCASMCs).

## Materials and Methods

### Proteoglycan cleavage assays

The recombinant human PGs, decorin (full length and glycosylated; 1–360 a.a., 0.5 ug, Abnova, Walnut, CA), biglycan (partial protein and glycosylated; 38–368 a.a., 1.5–5 µg, R&D Systems Minneapolis, MN) and betaglycan (partial protein and glycosylated; 21–781 a.a., 2.5–5 µg, R&D Systems) were incubated at room temperature for 24 h with 25–500 nM purified human GrB (Axxora, San Diego, CA). Reactions were carried out in 50 mM Tris buffer, pH 7.4. For inhibitor studies, GrB was incubated in the presence or absence of 200 µM of the serine protease inhibitor 3,4-dichloroisocoumarin (DCI; Santa Cruz Biotechnology Inc, Santa Cruz, CA) or inhibitor solvent control, dimethyl sulfoxide (DMSO; Sigma-Aldrich, St Louis, MO) for 4 h or 24 h. After incubation, proteins were denatured, separated on a 10% SDS-polyacrylamide gel and transferred to a nitrocellulose membrane. Ponceau stain (Fisher Scientific, Waltham, MA) was used to detect cleavage fragments.

### Cleavage assays on SMC-derived ECM

HCASMC (Clonetics/Lonza, Walkersville, MD), passages 3–7 were seeded in 6 well plates and once confluent, were incubated for 5 days in starvation media (smooth muscle growth medium (SMGM) +0.2% FBS) for adequate ECM synthesis. After incubation, cells were washed with PBS and incubated with 0.25 M ammonium hydroxide for 20 min, to remove cells but leave the ECM intact. The ECM was washed with water and incubated with PBS, GrB, GrB+DCI or GrB+DMSO for 24 h. Cleavage fragments were collected in the supernatant. ECM cleavage was detected by western blot for decorin or biglycan (primary antibodies from R&D Systems).

### N-terminal sequencing

For Edman degradation, 2–5 µg/lane of biglycan and betaglycan were incubated with 100–500 nM GrB for 24 h. Once run on a gel and transferred to a PVDF membrane, cleavage fragments were identified by Ponceau staining. The stain was removed by washes with distilled water and the membrane was dried and analyzed at the Advanced Protein Technology Center at the Hospital for Sick Kids (Toronto, ON).

### Michaelis-Menten kinetics

For Michaelis-Menten kinetics, 0.05–4 µM of decorin (in 50 mM Tris, pH 7.4), biglycan or betaglycan (in PBS, pH 7.4) was incubated with 100 nM GrB for 2.5 h at 37C. Reactions were stopped with SDS-PAGE loading buffer, run on an 8% gel and detected with G-250 Biosafe Coomassie Stain (Bio-Rad, Hercules CA). Proteoglycan cleavage was quantified by appearance of product for biglycan and betaglycan and loss of substrate for decorin. Enzyme kinetics calculations were carried out with GraphPad Prism software using linear regression curves for kinetics analysis.

### TGF-β1 release assays

TGF-β1 release assays were carried out using a method similar to that previously described for the assessment of MMP-induced TGF-β1 release from ECM [18]. Briefly, decorin, biglycan and betaglycan (15 µg/mL) were coated onto 48 well tissue culture plastic plates and allowed to incubate overnight at 4°C in PBS, pH 7.4. After blocking with 1% bovine serum albumin, 20 ng of active TGF-β1/well (Peprotech Inc, Rocky Hill, NJ) was added in dPBS containing calcium and magnesium (Invitrogen, Carlsbad, CA) for 5 h at RT. GrB, with or without DCI, was incubated on the wells and after 24 h, supernatants were removed, denatured, and run on a 15% SDS-PAGE gel. Nitrocellulose membranes were probed using a rat anti-human TGF-β1 antibody (BD Biosciences, Franklin Lakes, NJ) and IRDye® 800 conjugated affinity purified anti-rat IgG (Rockland Inc, Gilbertsville, PA). Bands were imaged using the Odyssey Infrared Imaging System (LI-COR Biotechnology, Lincoln, NE).

For TGF-β1 release from endogenous ECM, SMC-derived ECM was isolated as described above for the SMC-derived ECM cleavage assays. ECM was blocked with 1% BSA and incubated with 0.6 ng/ul TGF-β1 for 3 h at RT. Unbound TGF-β1 was washed away with PBS and GrB groups were incubated on the wells for 24 h. Supernatants, containing released TGF-β1, were run on a SDS-PAGE gel as described above.

### TGF-β1 bioavailability assays

For bioavailability assays, HCASMCs were seeded in 6 well plates in SmGM +5% fetal bovine serum (FBS, Invitrogen) and grown to confluence. At this time, cells were quiesced by serum removal for 24 h, after which 150 µl of release assay supernatants (as described above) at 5 ng/ml TGF-β1 positive control were added to the cells for 20 h. Cell lysates were assessed by SDS-PAGE/Western blotting for phosphorylated-SMAD-3 (p-SMAD3; Epitomics, Burlingame, CA) total SMAD-2/3 (t-SMAD-2/3; BD Biosciences) and the loading controls β-actin (Sigma-Aldrich) or β-tubulin (Millipore, Billerica, MA). Secondary IRDye® 800 conjugated antibodies (Rockland Inc) were utilized and imaged with the Odyssey Infrared Imaging System (LI-COR Biotechnology, Lincoln, Nebraska). Densitometric analysis was conducted on the Odyssey Infrared Imaging System and displayed graphically by p-SMAD-3/total-SMAD-3.

### Statistics

For densitometric analysis, a one way analysis of variance (ANOVA) with a Dunnett's post test was carried out and significance was determined at P<0.05.

## Results

### GrB cleaves decorin, biglycan and betaglycan

**Figure 1:**
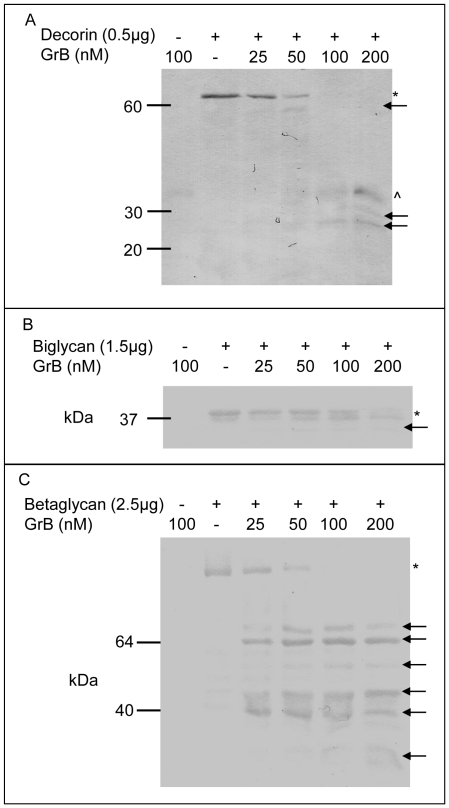
GrB-mediated cleavage of decorin, biglycan and betaglycan. Increasing concentrations of GrB (25, 50, 100 and 200 nM) were incubated with decorin (a), biglycan (b), and betaglycan (c) for 24 h at RT. * denotes full length protein, arrows indicate cleavage fragments and ∧ indicates GrB.

Incubation of decorin, biglycan and betaglycan with GrB resulted in the concentration-dependent generation of multiple cleavage fragments (Fig. 1a–c). Full length decorin (∼65 kDa) and 4 decorin cleavage fragments at ∼50 kDa and ∼30 kDa, were evident following GrB incubation. Biglycan was identified at ∼40 kDa, with cleavage fragments evident at ∼30 kDa and 20 kDa, while incubation of recombinant soluble betaglycan (∼100 kDa) with GrB resulted in multiple cleavage fragments at ∼60 kDa and 40 kDa. As all of these substrates are PGs and contain glycosaminoglycan (GAG) chains, the apparent MW of the full length proteins and fragments may not be accurate, as glycosylation can alter movement through the gel. As such, several of the proteins and protein fragments are observed as a smear as opposed to a condensed band.

**Figure 2:**
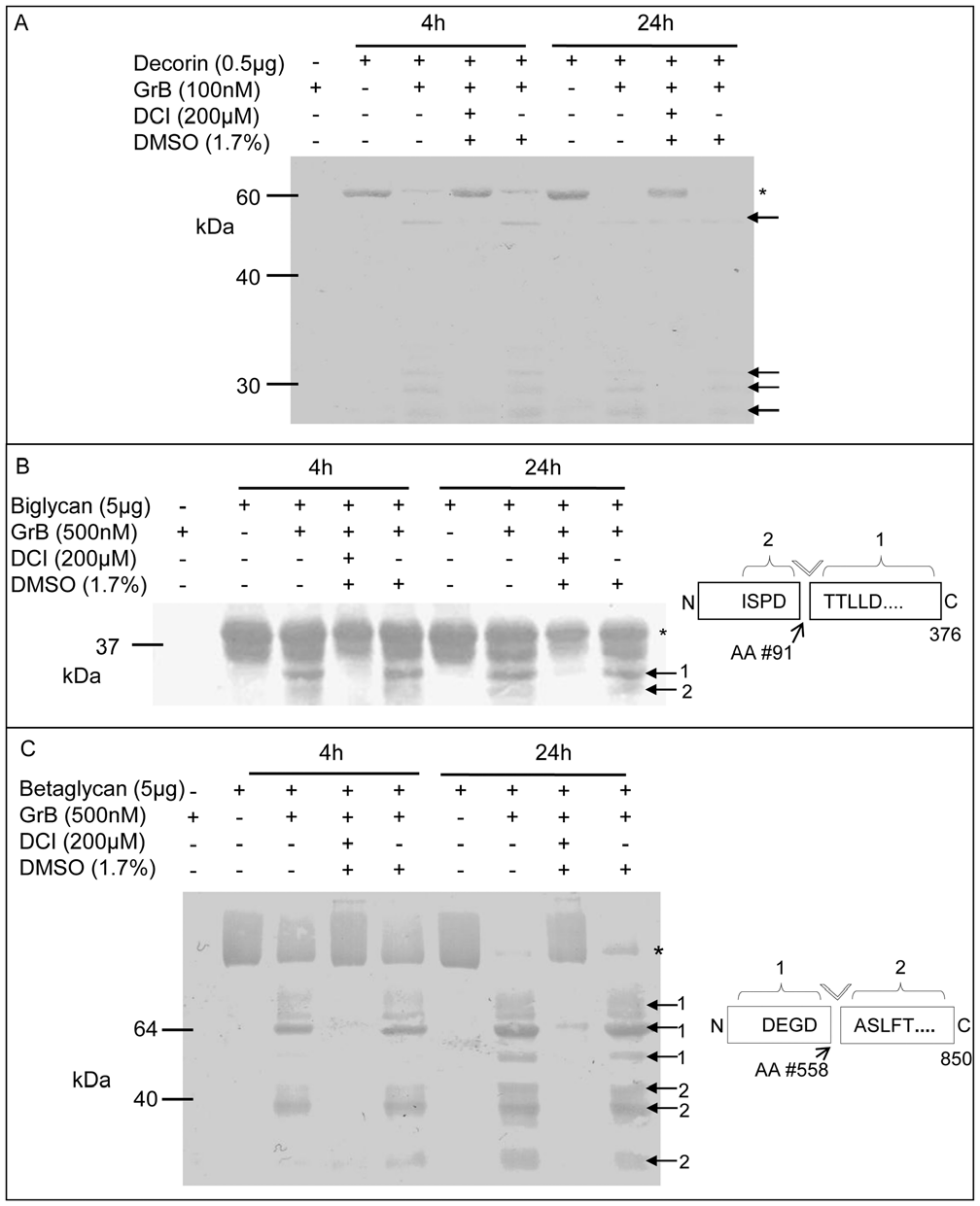
GrB-mediated PG cleavage is inhibited by DCI and cleavage site identification. GrB was incubated with decorin (a), biglycan (b) and betaglycan (c), +/− DCI and the solvent control DMSO, for 4 h and 24 h. Cleavage sites in biglycan and betaglycan were identified by N-terminal Edman degradation. * denotes full length protein, arrows indicate cleavage fragments, and cleavage sites are displayed on the right.

To confirm that decorin, biglycan and betaglycan proteolysis was mediated by GrB, DCI was included in reactions for 4 h or 24 h (Fig. 2a–c). Higher concentrations of PG substrates and GrB were utilized in this assay for favorable detection of cleavage fragments. DCI effectively inhibited decorin, biglycan and betaglycan cleavage at both time points while the vehicle control (DMSO) had no effect (Fig. 2a–c).

**Figure 3:**
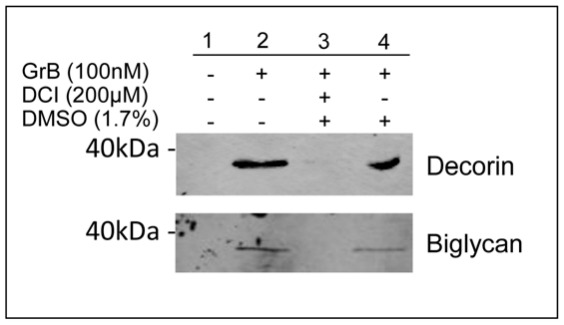
GrB cleaves native smooth muscle cell-derived decorin and biglycan. HCASMCs were incubated at confluency for adequate ECM synthesis. Cells were removed, GrB was incubated with the ECM and decorin and biglycan cleavage fragments were detected by western immunoblotting.

To verify these PGs are indeed GrB substrates in endogenous ECM, we repeated the cleavage assay with smooth muscle cell-derived ECM. As shown in Fig. 3, decorin and biglycan were indeed cleaved by GrB when associated with an endogenous ECM. Cleavage fragments for both proteins were apparent in the supernatant at around ∼32 kDa, the same cleavage fragment sizes seen in recombinant protein assays.

### GrB cleavage site identification

GrB cleavage sites were characterized in biglycan and betaglycan by Edman degradation (Fig. 2b–c). N-terminal sequence results for decorin were unable to be obtained due to low fragment yields, despite multiple trials. In biglycan, the cleavage site was identified at Ile-Ser-Pro-Asp^91^Thr-Thr-Leu-Leu-Asp, with a P1 residue of Asp (Fig. 2b). Interestingly, despite sequencing 6 unique bands for betaglycan, only one unique cleavage site was characterized, Asp-Glu-Gly-Asp^558^Ala-Ser-Leu-Phe-Thr, near the c-terminus of the protein (Fig. 2c). The n-terminal sequence results of betaglycan fragments labeled by ‘1’ corresponded to the n-terminus of the protein and the n-terminal sequence of fragments labeled with ‘2’ corresponded to the cleavage site (Fig. 2c).

### Michaelis-Menten kinetics

To determine if the cleavage of decorin, biglycan and betaglycan occurs at physiologically relevant rates, Michaelis-Menten kinetics was carried out on Commassie stained gels after 2 h incubation at 37C. Kcat/Km ratios for biglycan: 1.7×10^3^ M^−1^ s^−1^, betaglycan: 5.89×10^3^ M^−1^ s^−1^ and decorin: 1.0×10^3^ M^−1^ s^−1^.

### GrB-dependent cleavage of biglycan, decorin and betaglycan results in the release of active TGF-β1

**Figure 4:**
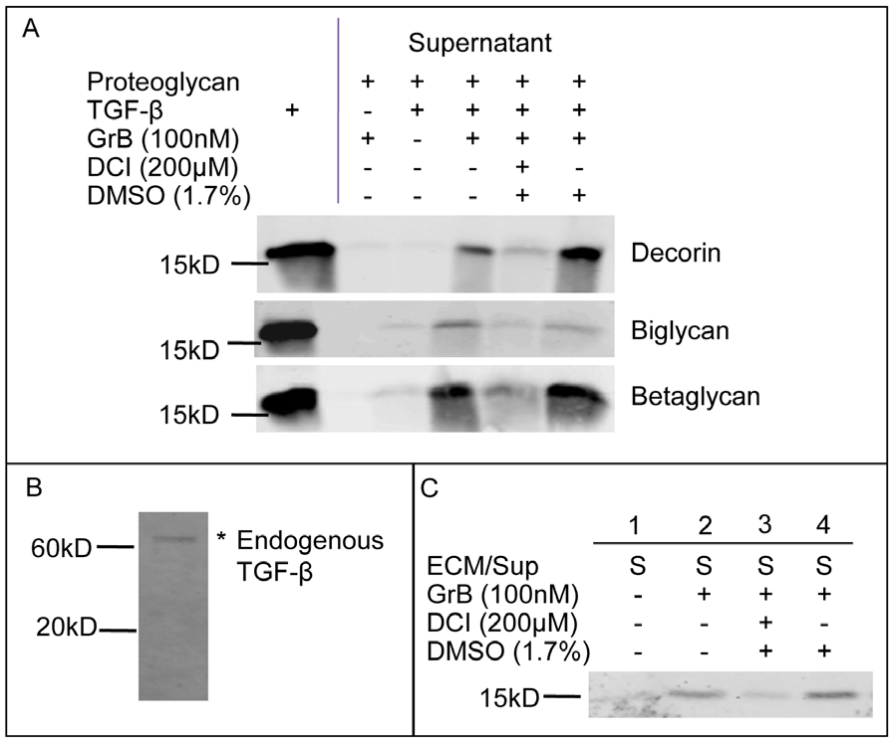
GrB-mediated cleavage of decorin, biglycan and betaglycan results in the release of active TGF-β1. Decorin, biglycan and betaglycan complexed with TGF-β1 were treated with GrB. Supernatants (containing released TGF-β1), were collected and released TGF-β1 was detected by Western blotting. Results shown are representative western blots from at least 3 separate experiments for each PG (a). As endogenous SMC-derived ECM only contains latent TGF-β (as shown in (b)), GrB-mediated release from active TGF-β1 supplemented ECM was also examined (c).

As decorin, biglycan and betaglycan sequester active TGF-β1 [22], [23], TGF-β1 release assays were performed to determine if GrB-mediated cleavage of these proteins resulted in active TGF-β1 release (Fig. 4a). Following 24 h of incubation, minimal TGF-β1 had dissociated from the plate in the absence of GrB, suggesting that the PG/TGF-β1 complexes were stable throughout the incubation time. After 24 h of GrB treatment, TGF-β1 was released into the supernatants, from all three PGs. This release was inhibited by DCI, suggesting the process was dependent on active GrB. TGF-β1 release was observed at GrB levels as low as 25 nM (Unpublished observations).

To confirm GrB can also release TGF-β from a heterogenous, endogenous ECM, SMC-derived ECM was utilized. However, in this closed *in vitro* setting TGF-β is present only as a latent complex, most likely due to a lack of extracellular activators in the culture system (Fig. 4b). As decorin, biglycan and betaglycan bind and sequester active TGF-β only, recombinant active TGF-β1 was supplemented on isolated ECM prior to GrB incubation. Upon GrB treatment, TGF-β1 was released from the ECM, while DCI prevented release (Fig. 4c). This suggests that GrB can release recombinant TGF-β1 from a native, heterogeneous ECM, in addition to recombinant PG matrices.

### TGF-β1 released by GrB remains active and induces SMAD signaling in smooth muscle cells

**Figure 5:**
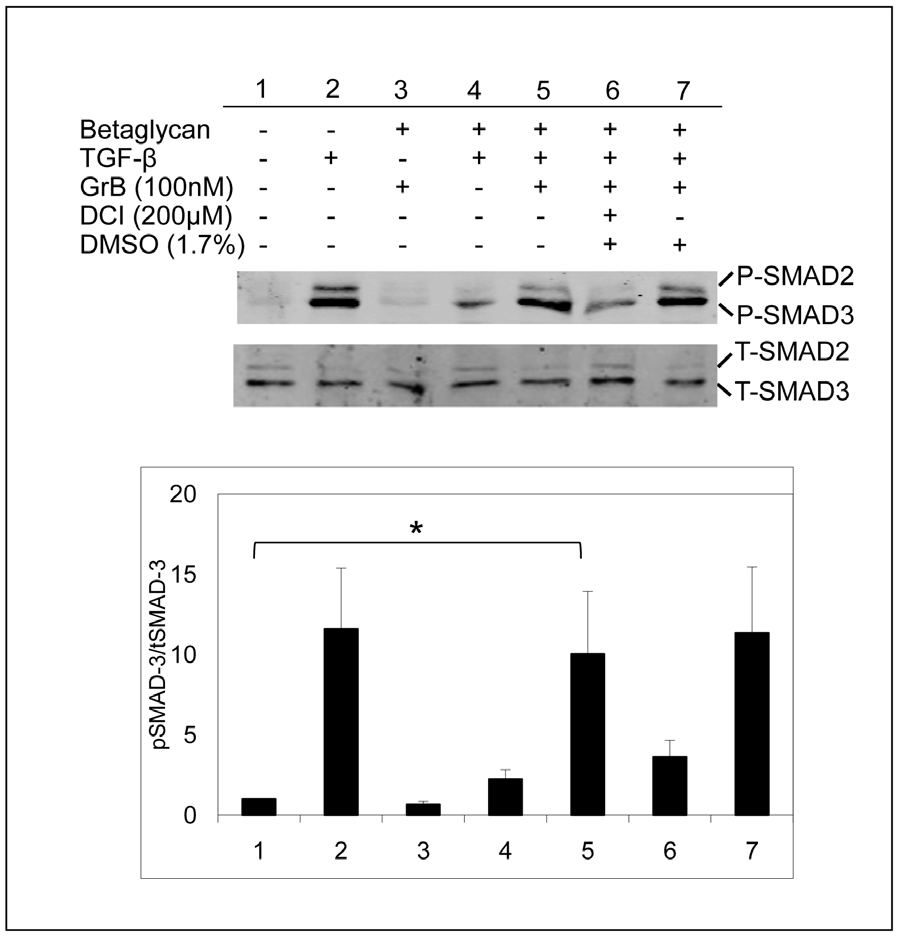
TGF-β1 released by GrB is active and induces SMAD-3 activation in HCASMCs. GrB+/−DCI was incubated on betaglycan/TGF-β1 complexes for 24 h. Supernatants (containing released TGF-β1) were added to HCASMC for 20 m and phosphorylated SMAD-2 and SMAD-3 levels were examined. TGF-β1 released by GrB is active and induces SMAD-3 signalling in HCASMCs (P<0.05). The result shown is representative of at least 5 experiments.

To determine that the TGF-β1 released by GrB remained active and was not bound to an inhibitory fragment, supernatants from the betaglycan release assay were incubated on HCASMC for 20 min (Fig. 5). TGF-β signaling was examined through the phosphorylation and activation of SMAD-3. HCASMC responded well to 5 ng/ml TGF-β1, with SMAD-3 phosphorylation observed at 20 min (P<0.05). The TGF-β1 released from betaglycan by GrB induced SMAD-3 signaling, confirming that it remained active (p<0.05). Total SMAD-3 levels also did not change.

## Discussion

Although GrB has been primarily studied in the context of its pro-apoptotic role in cytotoxic lymphocyte-mediated killing, several lines of evidence now imply an alternative, perforin-independent extracellular role for this protease in multiple chronic inflammatory diseases (Reviewed in [2]). In this study, we identify three novel extracellular substrates of GrB; decorin, biglycan and betaglycan. Furthermore we demonstrate that upon cleavage of these PGs by GrB, active TGF-β1 is released.

Approximately one third of GrB is released non-specifically into the extracellular milieu during immune cell engagement/degranulation and cytotoxic lymphocytes constitutively release GrB in the absence of target cell engagement [29], [30]. In further support of the biological relevance of non-specific release, the GrB released into the extracellular milieu during NK killing assays is sufficient to process IL-1α into a fragment with increased pro-inflammatory potential [31]. As such, even in cell culture studies there is sufficient leakage of GrB outside of cells to promote a biological response. Additionally, under certain conditions, non-inflammatory cell types (eg. Keratinocytes, chondrocytes, pneumocytes) can express and secrete GrB (reviewed in [3]). As many of these cell types do not express perforin or form immunological synapses, an extracellular role for GrB in pathogenesis is plausible. Indeed, in multiple chronic inflammatory conditions, GrB accumulates in extracellular fluids including plasma, cerebral spinal fluid, synovial fluid and bronchoalveolar lavage fluid (BALF, Reviewed in [2]). In addition, IL-1α fragments of similar size to GrB processed fragments were detected in BALF from patients with cystic fibrosis, chronic obstructive pulmonary disease and bronchiectasis [31]. Given that GrB is often detectable at levels 10 to 100-fold higher than normal in such fluids and retains its activity, it is highly probable that this protease could elicit a biological effect in the source tissues where its concentration would be expected to be significantly higher. In direct support of this, we have shown using both GrB and perforin knockout mice that GrB contributes to abdominal aortic aneurysm and skin aging through a perforin-independent mechanism involving ECM cleavage [8], [10]

Although the concentration range of GrB in inflamed tissues is presently unknown, the concentrations of GrB used in this study are likely to be physiologically relevant in chronic inflammatory disease. Mean levels of GrB in the plasma and synovial fluid of rheumatoid arthritis patients have been reported to be as high as 1 ng/ml and 3 ng/ml respectively (compared to <40 pg/ml in healthy patients), and similar increases of GrB in bodily fluids have been reported in other inflammatory diseases [5], [32]. Levels of circulating GrB would be expected to be several magnitudes lower than in source tissues, where GrB is being produced and released and where ECM cleavage is observed [2].

In this study, PG cleavage was detected at GrB concentrations as low as 25 nM, however, as the sensitivity of ponceau staining is relatively low, cleavage of these proteins may also occur at lower GrB concentrations. In addition to recombinant protein cleavage assays, we examined biglycan and decorin cleavage from a heterogeneous matrix synthesized by smooth muscle cells (SMCs). A ∼32 kDa cleavage fragment for both decorin and biglycan was detected in this assay, which were similar in size to the fragments derived from the recombinant substrate. As fragments were detected using monoclonal antibodies that recognize only one epitope of the proteins, other cleavage fragments were not detected. Soluble betaglycan was not detectable in ECM in this *in vitro* setting, suggesting it is either weakly expressed by SMCs or rarely shed by these cells *in vitro*.

GrB may also have affinity for ECM upon release, particularly in PG-rich ECM as GrB has been previously described to bind sulfated glycosaminoglycans. During the storage and release of GrB from cytotoxic granules, GrB binds to the heparan sulfate GAG chains of the PG serglycin [33]. Additionally, cells with reduced membrane GAG chain content displayed a decrease in GrB-mediated cell death, likely due to reduced electrostatic GrB transfer from serglycin to membrane associated GAG chains, such as chondroitin sulfate or heparan sulfate [34], [35], [36]. GrB affinity for these GAG chains has been proposed to be due to GrB cationic sites that bind electrostatically to anionic GAGs [35]. As decorin and biglycan contain chondroitin sulfate GAG chains and betaglycan contains both chondroitin sulfate and heparin sulfate chains, it is probable that GrB also exhibits increased affinity for these ECM PGs, potentially accumulating in PG-rich ECM.

Previously, GrB-mediated cleavage of another extracellular PG, aggrecan, was described [12] and other unidentified cartilage PGs, were shown to be cleaved in rheumatoid arthritis [37]. Whether or not decorin, biglycan or betaglycan were among the PGs cleaved in this study is not known but in our experience, it does appear that GrB has a preference for extracellular PG substrates.

In the present study, Michaelis-Menten kinetics was carried out to examine if GrB-mediated cleavage of these substrates was physiologically relevant in a biochemical context. In this environment, rates were lower than that for GrB-mediated cleavage of aggrecan, suggesting GrB may have a greater affinity for aggrecan than other proteoglycans [12]. It was also considerably lower than that determined for decorin cleavage by MMP 2, 3 and 7 [18]. However, biochemical assessment of *in vivo* relevance in an artificial test tube environment obviously has its limitations with respect to recapitulating other factors and what is actually occurring the extracellular milieu that is found in vivo. In the context of chronic diseases such as aneurysms and skin aging, this rate is likely significant as GrB has a high affinity for PGs leading to its increased accumulation and cleavage would occur over a prolonged period of time in areas of extracellular GrB accumulation, as suggested in previous publications [9], [10]. In addition, several other proteases have been characterized to cleave substrates as similar rates, and have been determined to be catalytically efficient [38], [39], [40], [41], [42]. Nonetheless, future studies are necessary to examine GrB-mediated proteoglycan cleavage *in vivo* and to identify GrB-derived cleavage fragments in chronic human disease.

Despite the fact that several extracellular GrB substrates have been identified, very few of the cleavage sites have been determined. In this study we define the GrB cleavage sites for biglycan and betaglycan. GrB residue preferences have been previously described, and GrB cleavage sites have generally been characterized with a P1 residue of aspartic acid [43], [44]. Less frequently however, cleavage at non-Asp P1 residues have been reported. The most commonly characterized alternate P1 residue is Glu acid but P1 residues of Ser, Asp and Met have also been shown but are suspected to occur at lower kinetics and may be nonspecific [45], [46]. In addition to its P1 specificity, GrB also has a preference for P3 residues that are negatively charged and P4 residues that are hydrophobic [45], [46]. P3, P2 and P1′ tended to be smaller residues, most likely due to the size restrictions of the peptide binding pocket [46]. In this study, we show that GrB cleaved these PG substrates at a P1 residue of Asp (biglycan: D^91^, betaglycan: D^558^), consistent with the literature described above. Biglycan contains a hydrophobic residue at P4 (Ile), betaglycan has a negative charge at P3 (Glu) and both contained relatively small resides in the P3, P2, P1′ area, reflecting the cleavage site trends described previously [46].

Interestingly, GrB is the only known extracellular protease capable of cleaving extracellular substrates at a P1 residue of aspartic acid. This unique specificity could potentially be used as a tool to initially screen for potential GrB-derived fragments in bodily fluids, of these substrates and others. In addition, the acidic side chain of aspartic acid is key for ionic interactions and molecular recognition by receptors. This is of particular interest for fragments containing RGD sequences, which have various biological activities in cell signaling and disease.

We have recently shown that GDKO mice are protected from the skin aging and frailty characteristic of aged apoE-KO mice and that GrB localization corresponds to areas of decorin degradation in apoE-KO mice [10]. The collagen fiber density loss evident in apoE-KO mice was not evident in GDKO mice, suggesting GrB-mediated degradation of decorin may result in collagen remodeling in aging skin. Importantly with respect to the present study, these studies support the premise that GrB-mediated PG cleavage does occur *in vivo*.

GrB contributes to aortic aneurysm via an extracellular mechanism involving the cleavage of the microfibrillar protein fibrillin-1 [8]. In a subsequent study, we demonstrate that adventitial decorin is reduced in apoE-KO aneurysms at sites adjacent to thrombi and in areas of injury [9]. GrB-deficiency and/or inhibition prevented this reduction in decorin leading to a thickened, healed, adventitia. Whether reduced decorin degradation leads to increased circumferential strength requires further elucidation, however, in the latter study a reduced incidence of rupture in GDKO mice compared to ApoE-KO mice was observed [8]. Of further interest, elevated GrB [8] and reduced decorin [47], [48] are observed in human aneurysm specimens. Adventitial collagen is critical for vessel strength/stability and collagen homeostasis/spacing is regulated by decorin, suggesting that decorin degradation would exert a negative impact on aortic wall strength and increase susceptibility to rupture [49].

A reduction in decorin levels has been found in wound healing models such as keloids, a variant of Ehlers-Danlos syndrome chronic skin ulcer and in the skin upon ultraviolet light exposure [50], [51], [52], [53]. Of particular interest, 32 kDa and 45 kDa decorin fragments were identified in human keloid tissue of similar size to GrB-derived fragments [51], [52].

Decorin, biglycan and betaglycan are known to sequester active TGF-β [22], [23]. Therefore, we asked whether cleavage of these PGs by GrB could release the growth factor. Matrix metalloproteases (MMP) are well characterized in their role in ECM degradation [54], [55] and cytokine/growth factor bioavailability as MMP-2, -3 and -7 have all been shown to release TGF-β1 from decorin [18]. To examine if GrB may have a similar effect we utilized a similar TGF-β1 release assay in our studies [18]. TGF-β1 was released from all three substrates. In addition, the TGF-β1 released by GrB induced SMAD-3 phosphorylation, confirming that GrB releases active TGF-β1 and does not alter TGF-β1 activity. As GrB liberates active TGF-β1 from the three most well described active TGF-β reservoirs, it may be a potent factor in influencing TGF-β bioavailability. To this end, we are not aware of another protease that has been described to release TGF-β from all three of these extracellular proteins. In addition, there are no confirmed extracellular inhibitors of GrB, as opposed to the MMPs, which are tightly-regulated extracellular proteases. One could speculate that GrB may not have an extracellular means of inhibition, as it appears to accumulate specifically during sustained inflammation. As such, increased extracellular GrB could lead to dysregulated TGF-β release and contribute to a multitude of deleterious effects at the site of injury. However, more work is necessary to confirm this hypothesis.

TGF-β down-regulates GrB and perforin expression in cytotoxic T cells [56]. However, the effect of TGF-β on GrB expression by other cell types is currently unknown. There is potential for TGF-β to act in a negative feedback loop in human tissue, whereby an increase in extracellular GrB levels would lead to an increase in TGF-β bioavailability. This could influence subsequent GrB expression, perhaps as a safety mechanism in disease. The effect of GrB-mediated release of TGF-β on subsequent GrB expression warrants further investigation.

In conclusion, the present knowledge of extracellular GrB activity in health and disease is in its infancy [57]. In addition to identifying three novel substrates for GrB, the present study provides further insight as to how an accumulation of GrB in the extracellular milieu could negatively impact and/or alter growth factor sequestration by the ECM.

## References

[1] Medema JP, Toes RE, Scaffidi C, Zheng TS, Flavell RA (1997). Cleavage of FLICE (caspase-8) by granzyme B during cytotoxic T lymphocyte-induced apoptosis.. Eur J Immunol.

[2] Boivin WA, Cooper DM, Hiebert PR, Granville DJ (2009). Intracellular versus extracellular granzyme B in immunity and disease: challenging the dogma.. Lab Invest.

[3] Hendel A, Hiebert PR, Boivin WA, Williams SJ, Granville DJ (2010). Granzymes in age-related cardiovascular and pulmonary diseases.. Cell Death Differ.

[4] Bratke K, Bottcher B, Leeder K, Schmidt S, Kupper M (2004). Increase in granzyme B+ lymphocytes and soluble granzyme B in bronchoalveolar lavage of allergen challenged patients with atopic asthma.. Clin Exp Immunol.

[5] Spaeny-Dekking EH, Hanna WL, Wolbink AM, Wever PC, Kummer AJ (1998). Extracellular granzymes A and B in humans: detection of native species during CTL responses in vitro and in vivo.. J Immunol.

[6] Young LH, Joag SV, Lin PY, Luo SF, Zheng LM (1992). Expression of cytolytic mediators by synovial fluid lymphocytes in rheumatoid arthritis.. Am J Pathol.

[7] Kondo H, Hojo Y, Tsuru R, Nishimura Y, Shimizu H (2009). Elevation of plasma granzyme B levels after acute myocardial infarction.. Circ J.

[8] Chamberlain CM, Ang LS, Boivin WA, Cooper DM, Williams SJ (2010). Perforin-independent extracellular granzyme B activity contributes to abdominal aortic aneurysm.. Am J Pathol.

[9] Ang LS, Boivin WA, Williams SJ, Zhao H, Abraham T (2011). Serpina3n attenuates granzyme B-mediated decorin cleavage and rupture in a murine model of aortic aneurysm.. Cell Death Dis.

[10] Hiebert PR, Boivin WA, Abraham T, Pazooki S, Zhao H (2011). Granzyme B contributes to extracellular matrix remodeling and skin aging in apolipoprotein E knockout mice.. Exp Gerontol.

[11] Gahring L, Carlson NG, Meyer EL, Rogers SW (2001). Granzyme B proteolysis of a neuronal glutamate receptor generates an autoantigen and is modulated by glycosylation.. J Immunol.

[12] Froelich CJ, Zhang X, Turbov J, Hudig D, Winkler U (1993). Human granzyme B degrades aggrecan proteoglycan in matrix synthesized by chondrocytes.. J Immunol.

[13] Buzza MS, Dyson JM, Choi H, Gardiner EE, Andrews RK (2008). Antihemostatic activity of human granzyme B mediated by cleavage of von Willebrand factor.. J Biol Chem.

[14] Mulligan-Kehoe MJ, Drinane MC, Mollmark J, Casciola-Rosen L, Hummers LK (2007). Antiangiogenic plasma activity in patients with systemic sclerosis.. Arthritis Rheum.

[15] Choy JC, Hung VH, Hunter AL, Cheung PK, Motyka B (2004). Granzyme B induces smooth muscle cell apoptosis in the absence of perforin: involvement of extracellular matrix degradation.. Arterioscler Thromb Vasc Biol.

[16] Buzza MS, Zamurs L, Sun J, Bird CH, Smith AI (2005). Extracellular matrix remodeling by human granzyme B via cleavage of vitronectin, fibronectin, and laminin.. J Biol Chem.

[17] Macri L, Silverstein D, Clark RA (2007). Growth factor binding to the pericellular matrix and its importance in tissue engineering.. Adv Drug Deliv Rev.

[18] Imai K, Hiramatsu A, Fukushima D, Pierschbacher MD, Okada Y (1997). Degradation of decorin by matrix metalloproteinases: identification of the cleavage sites, kinetic analyses and transforming growth factor-beta1 release.. Biochem J.

[19] Taylor AW (2009). Review of the activation of TGF-beta in immunity.. J Leukoc Biol.

[20] Schiller M, Javelaud D, Mauviel A (2004). TGF-beta-induced SMAD signaling and gene regulation: consequences for extracellular matrix remodeling and wound healing.. J Dermatol Sci.

[21] Dallas SL, Miyazono K, Skerry TM, Mundy GR, Bonewald LF (1995). Dual role for the latent transforming growth factor-beta binding protein in storage of latent TGF-beta in the extracellular matrix and as a structural matrix protein.. J Cell Biol.

[22] Lopez-Casillas F, Payne HM, Andres JL, Massague J (1994). Betaglycan can act as a dual modulator of TGF-beta access to signaling receptors: mapping of ligand binding and GAG attachment sites.. J Cell Biol.

[23] Hildebrand A, Romaris M, Rasmussen LM, Heinegard D, Twardzik DR (1994). Interaction of the small interstitial proteoglycans biglycan, decorin and fibromodulin with transforming growth factor beta.. Biochem J.

[24] Fleischmajer R, Fisher LW, MacDonald ED, Jacobs L, Perlish JS (1991). Decorin interacts with fibrillar collagen of embryonic and adult human skin.. J Struct Biol.

[25] Schonherr E, Witsch-Prehm P, Harrach B, Robenek H, Rauterberg J (1995). Interaction of biglycan with type I collagen.. J Biol Chem.

[26] Velasco-Loyden G, Arribas J, Lopez-Casillas F (2004). The shedding of betaglycan is regulated by pervanadate and mediated by membrane type matrix metalloprotease-1.. J Biol Chem.

[27] Arribas J, Borroto A (2002). Protein ectodomain shedding.. Chem Rev.

[28] Mythreye K, Blobe GC (2009). Proteoglycan signaling co-receptors: roles in cell adhesion, migration and invasion.. Cell Signal.

[29] Isaaz S, Baetz K, Olsen K, Podack E, Griffiths GM (1995). Serial killing by cytotoxic T lymphocytes: T cell receptor triggers degranulation, re-filling of the lytic granules and secretion of lytic proteins via a non-granule pathway.. Eur J Immunol.

[30] Prakash MD, Bird CH, Bird PI (2009). Active and zymogen forms of granzyme B are constitutively released from cytotoxic lymphocytes in the absence of target cell engagement.. Immunol Cell Biol.

[31] Afonina IS, Tynan GA, Logue SE, Cullen SP, Bots M (2011). Granzyme B-dependent proteolysis acts as a switch to enhance the proinflammatory activity of IL-1alpha.. Mol Cell.

[32] Tak PP, Spaeny-Dekking L, Kraan MC, Breedveld FC, Froelich CJ (1999). The levels of soluble granzyme A and B are elevated in plasma and synovial fluid of patients with rheumatoid arthritis (RA).. Clin Exp Immunol.

[33] Raja SM, Wang B, Dantuluri M, Desai UR, Demeler B (2002). Cytotoxic cell granule-mediated apoptosis. Characterization of the macromolecular complex of granzyme B with serglycin.. J Biol Chem.

[34] Raja SM, Metkar SS, Honing S, Wang B, Russin WA (2005). A novel mechanism for protein delivery: granzyme B undergoes electrostatic exchange from serglycin to target cells.. J Biol Chem.

[35] Bird CH, Sun J, Ung K, Karambalis D, Whisstock JC (2005). Cationic sites on granzyme B contribute to cytotoxicity by promoting its uptake into target cells.. Mol Cell Biol.

[36] Veugelers K, Motyka B, Goping IS, Shostak I, Sawchuk T (2006). Granule-mediated killing by granzyme B and perforin requires a mannose 6-phosphate receptor and is augmented by cell surface heparan sulfate.. Mol Biol Cell.

[37] Ronday HK, van der Laan WH, Tak PP, de Roos JA, Bank RA (2001). Human granzyme B mediates cartilage proteoglycan degradation and is expressed at the invasive front of the synovium in rheumatoid arthritis.. Rheumatology (Oxford).

[38] Ito S, Taguchi H, Hamada S, Kawauchi S, Ito H (2008). Enzymatic properties of cellobiose 2-epimerase from Ruminococcus albus and the synthesis of rare oligosaccharides by the enzyme.. Appl Microbiol Biotechnol.

[39] Kratzer R, Pukl M, Egger S, Vogl M, Brecker L (2011). Enzyme identification and development of a whole-cell biotransformation for asymmetric reduction of o-chloroacetophenone.. Biotechnol Bioeng.

[40] Bae YA, Kim SH, Lee EG, Sohn WM, Kong Y (2011). Identification and biochemical characterization of two novel peroxiredoxins in a liver fluke, Clonorchis sinensis.. Parasitology.

[41] Chau Y, Tan FE, Langer R (2004). Synthesis and characterization of dextran-peptide-methotrexate conjugates for tumor targeting via mediation by matrix metalloproteinase II and matrix metalloproteinase IX.. Bioconjug Chem.

[42] Ling HB, Wang GJ, Li JE, Tan HR (2008). sanN encoding a dehydrogenase is essential for Nikkomycin biosynthesis in Streptomyces ansochromogenes.. J Microbiol Biotechnol.

[43] Casciola-Rosen L, Garcia-Calvo M, Bull HG, Becker JW, Hines T (2007). Mouse and human granzyme B have distinct tetrapeptide specificities and abilities to recruit the bid pathway.. J Biol Chem.

[44] Kaiserman D, Bird CH, Sun J, Matthews A, Ung K (2006). The major human and mouse granzymes are structurally and functionally divergent.. J Cell Biol.

[45] Harris JL, Peterson EP, Hudig D, Thornberry NA, Craik CS (1998). Definition and redesign of the extended substrate specificity of granzyme B.. J Biol Chem.

[46] Van Damme P, Maurer-Stroh S, Plasman K, Van Durme J, Colaert N (2009). Analysis of protein processing by N-terminal proteomics reveals novel species-specific substrate determinants of granzyme B orthologs.. Mol Cell Proteomics.

[47] Tamarina NA, Grassi MA, Johnson DA, Pearce WH (1998). Proteoglycan gene expression is decreased in abdominal aortic aneurysms.. J Surg Res.

[48] Mohamed SA, Sievers HH, Hanke T, Richardt D, Schmidtke C (2009). Pathway analysis of differentially expressed genes in patients with acute aortic dissection.. Biomark Insights.

[49] Danielson KG, Baribault H, Holmes DF, Graham H, Kadler KE (1997). Targeted disruption of decorin leads to abnormal collagen fibril morphology and skin fragility.. J Cell Biol.

[50] Gambichler T, Tomi NS, Skrygan M, Altmeyer P, Kreuter A (2007). Significant decrease of decorin expression in human skin following short-term ultraviolet exposures.. J Dermatol Sci.

[51] Mukhopadhyay A, Wong MY, Chan SY, Do DV, Khoo A (2010). Syndecan-2 and decorin: proteoglycans with a difference–implications in keloid pathogenesis.. J Trauma.

[52] Carrino DA, Mesiano S, Barker NM, Hurd WW, Caplan AI (2012). Proteoglycans of uterine fibroids and keloid scars: similarity in their proteoglycan composition.. Biochem J.

[53] Wu J, Utani A, Endo H, Shinkai H (2001). Deficiency of the decorin core protein in the variant form of Ehlers-Danlos syndrome with chronic skin ulcer.. J Dermatol Sci.

[54] Amalinei C, Caruntu ID, Balan RA (2007). Biology of metalloproteinases.. Rom J Morphol Embryol.

[55] Chang C, Werb Z (2001). The many faces of metalloproteases: cell growth, invasion, angiogenesis and metastasis.. Trends Cell Biol.

[56] Thomas DA, Massague J (2005). TGF-beta directly targets cytotoxic T cell functions during tumor evasion of immune surveillance.. Cancer Cell.

[57] Granville DJ (2010). Granzymes in disease: bench to bedside.. Cell Death Differ.

