# Correction: Granzyme B Cleaves Decorin, Biglycan and Soluble Betaglycan, Releasing Active Transforming Growth Factor-β1

**DOI:** 10.1371/annotation/b1e4ff60-ba18-4f92-b856-0f2dd27e9a65

**Published:** 2012-05-10

**Authors:** Wendy A. Boivin, Marlo Shackleford, Amanda Vanden Hoek, Hongyan Zhao, Tillie L. Hackett, Darryl A. Knight, David J. Granville

There were errors in the published Author Contributions. The correct Author Contributions are: Conceived and designed the experiments: WAB AV TLH DJG. Performed the experiments: WAB MS HZ. Analyzed the data: WAB AV TLH DAK DJG. Contributed reagents/materials/analysis tools: TLH DAK DJG. Wrote the paper: WAB. 

